# Curcumin: A Potential Candidate in Prevention of Cancer via Modulation of Molecular Pathways

**DOI:** 10.1155/2014/761608

**Published:** 2014-09-10

**Authors:** Arshad H. Rahmani, Mohammad A. Al Zohairy, Salah M. Aly, Masood A. Khan

**Affiliations:** ^1^Department of Medical Laboratories, College of Applied Medical Sciences, Qassim University, Buraida, Saudi Arabia; ^2^Department of Pathology, Faculty of Vet. Medicine, Suez Canal University, Ismailia, Egypt; ^3^Department of Basic Health Science, College of Applied Medical Sciences, Qassim University, Saudi Arabia

## Abstract

Cancer is the most dreadful disease worldwide in terms of morbidity and mortality. The exact cause of cancer development and progression is not fully known. But it is thought that cancer occurs due to the structural and functional changes in the genes. The current approach to cancer treatment based on allopathic is expensive, exhibits side effects; and may also alter the normal functioning of genes. Thus, a safe and effective mode of treatment is needed to control the cancer development and progression. Some medicinal plants provide a safe, effective and affordable remedy to control the progression of malignant cells. The importance of medicinal plants and their constituents has been documented in Ayurveda, Unani medicine, and various religious books. Curcumin, a vital constituent of the spice turmeric, is an alternative approach in the prevention of cancer. Earlier studies have shown the effect of curcumin as an antioxidant, antibacterial, antitumor and it also has a noteworthy role in the control of different diseases. In this review, we summarize the understanding of chemopreventive effects of curcumin in the prevention of cancer via the regulation of various cell signaling and genetic pathways.

## 1. Introduction

Population growth and aging may contribute to a dramatic increase in the numbers of cancer cases. The exact cause of the cancer development and progression is still not well known. But it is thought to be as a result from alterations in the various genetic [1–3] and metabolic pathways. The present regime to cancer treatment, based on synthetic drugs and chemotherapy/radiotherapy, is expensive and also alters the various mechanisms of the normal actions of genes. Presently, several medicinal plants and their constituents are in use to manage the development and progression of various diseases and have been found effective, safe, and less expensive. The importance of medicinal plants has been discussed in different religious books including Christianity, Hinduism, and Islam. Our Prophet Mohammad (PBUH) used and recommended various plants and their products in the cure of diseases [4, 5]. In the present scenario, various parts of the world are using different types of local plants or products like turmeric in Indian cuisine and oregano in Italy, olive in Spain, and Ajwa dates in Saudi Arabia in the treatment and prevention of various diseases. Earlier studies have reported that olive, dates, and black seed show role in cancer prevention through modulation of various activities [6–8].

**Figure 1:**
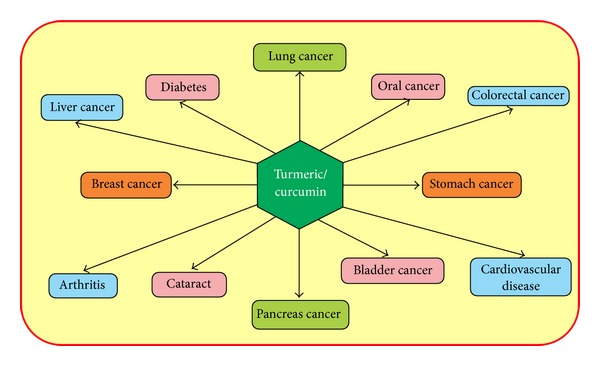
Turmeric/curcumin shows pivotal role in the prevention of diseases including cancer.

*Curcumin* is a polyphenolic compound derived from the popular Indian spice turmeric plant. It is a member of the Zingiberaceae (ginger) family, which is native to Southeast Asia [9], chemical structure was characterized in 1910 by Milobedeska and colleagues, and synthesis was confirmed by Lampe and colleagues in 1913 [10, 11].* Curcumin* is lipophilic in nature which shows low solubility and stability in aqueous solution. It is extensively used in Ayurveda, Unani, Siddha, and Chinese medicine for the management of various diseases such as wound, inflammation, and cancer (Figure 1) and used in curries and dishes especially in spicy dishes in India, Pakistan, Bangladesh, and other countries of Asia. Curcumin, because of its special properties such as being antiprotozoal and antioxidant and this uniqueness, might have a significant effect on several types of diseases anticipation [12, 13]. Important reviews based on curcumin provide an overview of the history, chemistry, analogs, and mechanism of action of curcumin [14] and another study discussed in detail the therapeutic implications of curcumin in patients with pancreatic cancer [15].

## 2. Curcumin: Modulator of Molecular Pathways

Tumorigenesis and tumor progression are thought to be as a result of some changes in the different types of genetic pathways [16, 17]. Curcumin, chief constituents of turmeric, shows a vital role in cancer prevention and treatment through modulation of various biological activities including molecular cascades. However, understanding the turmeric's mechanism of action in the activation or inactivation of genetic pathways will provide significant information to develop therapeutic approaches to manage various types of cancers.

### 2.1. Effect of Curcumin on Tumor Suppressor Genes

Tumor suppressor genes play a vital and significant role in the inhibition of cancer formation and its progression. When an alteration or mutation occurs in a gene, then tumor suppressor gene lost its ability to perform normal function. Tumor suppressor gene p53 is the guardian of all genes and regulates the various cellular and molecular pathways and prevents cancer formation. Numerous* in vivo* and* in vitro* reports showed that turmeric and its constituents have a significant role in cancer prevention or inhibition. An important study showed that curcumin down-regulates the expression of p53, as well as the survival genes egr-1, c-myc and bcl-XL in B cells [18]. Another report has also indicated that curcumin inhibits cell cycle progression of immortalized human umbilical vein endothelial cells via upregulating the CDK inhibitors p21WAF1/CIP1, p27KIP1, and p53 [19]. Further studies reported that curcumin mainly acts in p53-dependent manner and also showed that wild p53 was highly susceptible to curcumin toxicity [20].

Another tumor suppressor gene, phosphatase and tensin homolog deleted on chromosome ten (PTEN) has a role in the progression of the cell cycle and apoptosis. The alteration or mutation of PTEN gene has been noticed in several types of cancers. A study of the curcumin has shown that PTEN increases the curcumin-induced apoptosis, whereas inactive PTEN decreases/inhibits the curcumin-induced apoptosis [21]. A study showed that difluorinated curcumin (CDF), a nontoxic analog, modulates the expression of miR-21 and PTEN in pancreatic cancer [22–25].

The retinoblastoma is a type of tumour suppressor gene and shows an important role in the control of cell cycle. pRb, the protein coded for by the* RB1* gene, shows an important role in cell cycle regulation, promoting G1/S arrest and growth restriction via inhibition of the E2F transcription factors [26]. It is inactivated through hyperphosphorylation catalyzed by the cyclin D-cyclin-dependent kinase 4 (cyclin D-cdk4) and cyclin E-cdk2 complexes [27–29]. Various medicinal plants and their constituents show a vital role in the regulation of Rb genes via regulation of phophorylation. In this vista, curcumin, chief constituents of turmeric also shows an important role in modulation of Rb gene via reduction in hyperphosphorylation. An important study based on prostate cancer cells has revealed that curcumin induced the expression of cyclin-dependent kinase (CDK) inhibitors p16, p21, and p27 and inhibited the expression of cyclin E and cyclin D1 and hyperphosphorylation of retinoblastoma (Rb) protein [30] and another study has shown that suppression of cyclin D1 by curcumin led to inhibition of CDK4-mediated phosphorylation of retinoblastoma protein [31].

### 2.2. Effect of Curcumin on Apoptotic Genes

**Figure 2:**
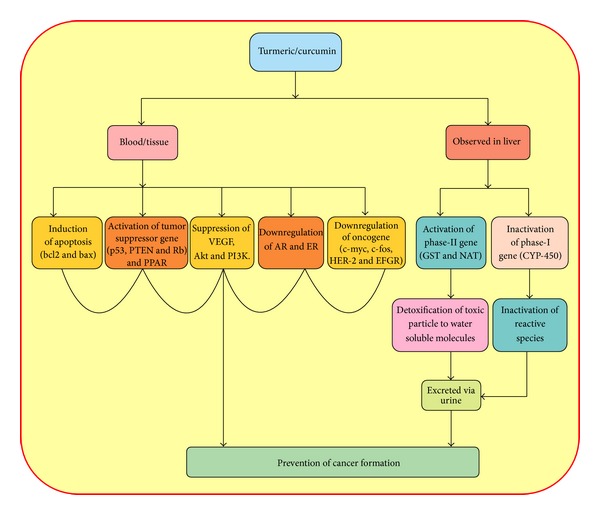
Turmeric/curcumin shows an important role in cancer prevention via induction of apoptosis, activation of tumour suppressor gene and phase II gene, and inactivation of oncogene, hormonal receptor gene, and angiogenesis and phase-I gene.

Apoptosis is one of the prerequisites to maintain the normal and healthy internal milieu. Any alteration or change in the normal process of apoptosis may increase cell survival and support the tumor development and progression [32, 33]. Curcumin plays a vital role in the upregulation of different proapoptotic genes and at the same time downregulates some of the antiapoptotic genes and by this way balances the apoptosis process (Figure 2). An interesting study showed that curcumin induces apoptosis in scleroderma lung fibroblasts (SLF) without affecting normal lung fibroblasts [34]. Furthermore, curcumin has shown an antitumor activity and was involved in the apoptosis induction and the modulation of key apoptotic proteins such as Bax and bcl-2 [35]. A study has reported that growth arrest and apoptosis of B cell lymphoma occur through the down regulation of c-myc, bcl-XL, and p53 with the treatment of curcumin [36]. Another report in human breast cancer cell line showed that CD437 induces G0-G1 arrest and apoptosis via regulation of p21WAFI/CIPI, Bcl-2, and Bax in a p53 independent manner [37]. Another study on p53-null cells, as well as TR9-7 cells, reported that curcumin induces apoptosis in tumor cells via a p53-dependent pathway and Bax act as downstream effectors of p53 [38].

*Curcumin* induces apoptosis in a range of tumor cell lines through activation of caspase-3, cytochrome c release, and downregulation of bcl-2 [39–42].* Curcumin* has shown an apoptotic effect by inhibiting various genes such as protein tyrosine kinase, protein kinase C, c-myc mRNA expression, and bcl-2 mRNA expression [43] and also mitochondrial pathway. Earlier studies have shown that curcumin possesses an apoptotic activity in different types of cancer cell such as human colon cancer cells, stomach, and skin tumors, breast cancer cells, and prostate cancer cells [44–47]. Study of colon cancer cell line showed that apoptosis was increased in response to curcumin [48, 49]. Curcumin also showed a vital role in decreasing cell proliferation in a dose dependent manner [48–50]. Curcumin may lower the incidence of various cancers, including urothelial malignancies [51, 52] and also may induce apoptosis in MBT-2 cells [53] and G2/M arrest of T24 cells [54]. Experimental studies showed that the downregulation of the expression of antiapoptotic protein occurs with curcumin treatment [55, 56].

### 2.3. Effect of Curcumin on Angiogenesis

Angiogenesis is a complex process involving widespread interaction between the cells, soluble factors, and ECM components [57]. It also shows a vital effect in tumor growth and is triggered by chemical signals from tumor cells in a phase of rapid growth [58]. There are several angiogenic factors such as vascular endothelial growth factor (VEGF), basic fibroblast growth factor (bFGF), angiogenin, transforming growth factor (TGF-α, TGF-*β*), and epidermal growth factor. These factors show a critical role in tumor angiogenesis via cancerous tumor cells by releasing molecules and sending signals to surrounding normal host tissue [59]. VEGF is a crucial survival factor for endothelial cells in the process of physiological, tumor angiogenesis and induces the expression of antiapoptotic proteins in the endothelial cells [60]. There are certain drugs, like Bevacizumab (Avastin), available as an inhibitor of VEGF action in the treatment of cancer. These drugs are expensive and a large group of the population cannot afford their cost. However, a safe and affordable natural product is needed to control the cancer development.

Earlier studies have shown that curcumin is an inhibitor of VEGF in different types of cancer, including orthotopically implanted pancreatic tumors [61]. Important record via* in vitro* and* in vivo* studies showed that curcumin suppresses the proliferation of human vascular endothelial cells and also abrogates the FGF-2-induced angiogenic response [62, 63]. Moreover, curcumin has the ability to inhibit both VEGF and its receptor in various cancer types; it might be useful as an antiangiogenic agent [64, 65]. Besides, curcumin plays a major role in the suppression of transcriptional activity of AP62 and HIF-1 and causes a reduction in the expression of VEGF [66]. An important study results suggested that curcumin potentiates the antitumor effects of gemcitabine in pancreatic cancer via suppressing proliferation, angiogenesis, NF-*κ*B, and NF-*κ*B-regulated gene products [61]. A study of adenoid cystic carcinoma cells showed that curcumin significantly inhibited the growth, survival, migration/invasion, and downregulates VEGF and MMP-2/9 or inhibits the mTOR and NF-*κ*B pathways [67].

### 2.4. Effect of Curcumin on Phase I and Phase II Genes/Enzymes

Xenobiotics are molecules introduced into the body from the environment and not produced inside the body. The body then metabolizes them through two phases of transformation: Phase I and Phase II.

The turmeric has shown a significant effect on the regulation of xenobiotic metabolism via inhibition of the phase I and activates the phase II gene/enzymes. In the phase I reactions, addition of a functional polar group normally results in a relatively small increase in hydrophilicity and may cause metabolic activation. Cytochrome P450 (CYP) is the main enzymes in phase I and shows the vital effect on the activation of carcinogens. So, control of CYP450 activity is the main issue in cancer prevention through increasing the degree of cellular safety. A study in rat model showed that curcumin inhibits the alkylation reaction catalyzed by CYP1A1, 1A2 [68]. In another interesting study, it was reported that CYP plays a vital role in the formation of aflatoxine-DNA adduct and this intermediate product is suppressed or inhibited by curcumin treatment [69]. Curcumin has shown its effect in hampering CYP1A1 activity in DMBA-treated cells and also inhibited the metabolic activation of DMBA and decreased the DMBA-induced cytotoxicity [70].

In the Phase II reactions, conjugation with a small hydrophilic endogenous substance increases the hydrophilicity and facilitates the emission. However, the activation of Phase II enzymes such as Glutathione S Transferase in the treatment and suppression of cancer is critical and is a significant strategy. Several earlier studies reported that turmeric and its constituents play a significant role in the prevention of cancer via the activation of GST genes.

An important finding showed that turmeric/curcumin enhances the activity of Phase II enzyme GST [71–76]. Curcumin also elevates the protein as well as mRNA expressions of GSTs and NQO1 in mouse tissues, suggesting a role of curcumin in transcriptional regulation of phase II enzymes [77]. Moreover, curcumin induces GST expression by signalling through the nuclear erythroid-derived 2-related factor 2 (NRF-2) and NF-*κ*B via an antioxidant response element [78].

### 2.5. Androgen Degradation/Down Regulation by Curcumin

The androgen receptor (AR) is a ligand activated steroid hormone receptor that plays a vital and significant role in developing the function of normal prostrate as well as in prostate cancer development and progression [79, 80]. Change in the function or overexpression of AR has been observed in cancer [81]. The treatment basis on allopath like hormone therapy was considered as a potential treatment, but its limitation/disadvantage as prostate cancer cells become progressive and may lead to metastasis [82]. The regulation of AR activities is a critical step in the control or suppression of tumor development and progression. An important study of curcumin on androgen dependent LNCaP prostate cancer cell line and an androgen independent PC-3 prostate cancer cell showed that AR protein level is downregulated [83]. Earlier studies had shown that curcumin downregulates the transactivation and expression of AR and AR-related cofactors [84]. The constituent has a potential therapeutic effect on prostate cancer cells through the downregulation of AR and AR-related cofactors AP-1, NF, and CBP [84]. Another study reported that curcumin acts as an inducer of apoptosis in both androgen-dependent and hormone refractory prostate cancer cells [85]. Curcumin blocks the activation of androgen and IL-6 on prostate-specific antigen expression in human prostatic carcinoma cells [86].

### 2.6. Effect of Curcumin on PI3 K/Akt Pathways

PI3 K/Akt signalling pathway is important and critical in mediating cell survival, proliferation, migration, and angiogenesis. PI3 K catalyzes the production of the lipid secondary messenger phosphatidylinositol-3,4,5-triphosphate including the serine/threonine kinase Akt [87, 88]. Mutation and/or loss of PTEN function plays an important role in the activation of PI3 K and is associated with the growth and progression of various types of cancers [89, 90]. Activated PI3 K shows a role in the conversion of phosphatidylinositol into PtdIns(3,4)P2 (PIP2) and PtdIns(3,4,5)P3 (PIP3). Phosphatidylinositol dependent kinases 1/2 (PDKs 1/2) play a key role in the phosphorylation of Akt at residues Thr308 and Ser473 [91–93]. Activated Akt plays a role in promoting cell survival by suppressing apoptosis via subsequent modulation of a wide range of target molecules [94–97]. PTEN, a tumor suppressor gene, is a multifunctional phosphatase whose major substrate is phosphatidylinositol-3,4,5-trisphosphate (PIP3) [98]. Phosphatase activity of PTEN plays an important effect in dephosphorylation of PIP3. By this way PTEN negatively regulates the phosphoinositide-3-kinase (PI3 K)-PKB/Akt pathway and prevents the tumor development or tumor suppression. The inhibition of the PI3 K/Akt and activation of PTEN pathway is a good strategy in the prevention of cancer. An important study showed that curcumin inhibits the phosphorylation of Akt, mTOR, and their downstream substrates, and this inhibitory effect acts downstream of phosphatidylinositol 3-kinase and phosphatidylinositol-dependent kinase1 [99].

### 2.7. Effect of Curcumin on Cycloxygenase Enzyme

**Figure 3:**
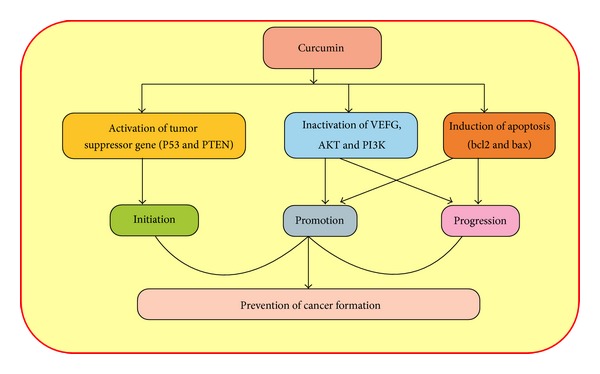
Curcumin shows an important role in the prevention of cancer through the inhibition of initiation, promotion, and progression steps via modulation of molecular cascades.

COX is an inducible enzyme in the conversion of arachidonic acid to prostaglandins (PGs). There are two types of cycloxygenase: COX1 plays a vital role in physiological functions and COX2 is upregulated or overexpressed in various types of cancers [100–102]. It was previously stated that curcumin inhibits the critical stage of tumor initiation and promotion stages [103, 104] and COX inhibition [105]. Curcumin also inhibits the COX2 expression on colon cancer cell lines [106]. Earlier studies reported that curcumin plays an important role in the downregulation of the expression of COX-2 and finally prevents or suppresses the cancer progression [107]. Moreover, curcumin plays a significant role in the cancer prevention via controlling the activities of various genes in the initiation, promotion, and progression stage of tumor development and progression (Figure 3).

### 2.8. NF-*κ*B and Curcumin in Cancer Prevention

NF-*κ*B family of transcription factors shows an important role in immune, inflammatory response and also stimulates the development and progression of cancer. In this regard, an important study demonstrated that curcumin showed as an anticancer, antioxidant, and anti-inflammatory effect via the downregulation of the transcription factors NF-*κ*B, AP-1, and Egr-1 [108] and repression of the genes for cell adhesion molecules (chemokines, TNF, Cox-2, and MMP-9) [109, 110]. Another study showed that curcumin is a pharmacologically safe agent and has been involved in the suppression of NF-*κ*B activation and NF-*κ*B gene products [111].

An important study in pancreatic cancer cells reported that curcumin showed a vital role in the suppression of NF-*κ*B activation by inhibiting I*κ*B kinase, ultimately induces I*κ*Bα phosphorylation, and inhibits the NF-*κ*B downstream gene expression [112].

Several findings showed that curcumin suppresses the expression of a variety of NF-*κ*B regulated gene products involved in cancer development and progression such as cyclin D1, VEGF, COX-2, c-myc, Bcl-2, ICAM-1, and MMP-9 [110, 113–115].

Numerous studies has shown that curcumin is a potent inhibitor of NF-*κ*B activation [63, 109, 111, 116–119].

### 2.9. Effect of Curcumin on Oncogene

Alteration or mutation of protooncogene is key factors in the development and progression of various types of tumours. An activated oncogene has been noticed in various types of cancer [120–122]. Safe route of inactivation of an oncogene is a prime interest in the prevention of tumor. Several earlier investigations reported that curcumin shows a significant effect in cancer prevention via the inactivation of oncogene. Curcumin downregulated N-Myc [123] in various cancer types and decreased the expression of proto-oncogenes such as* ras* and* fos* in tumorous skin [124]. A report on the effect of curcumin in hepatocellular carcinoma revealed that curcumin blocked transactivation of the c-Met promoter through AP-1 [125]. Another finding on curcumin effect in the downregulation of oncogene showed that curcumin induced the antiproliferative, antimigratory and apoptotic effects via the downregulation of various genes, including c-Myc, N-Myc, cyclin D1, and antiapoptotic factors Bcl-2 and Bcl-xL [126]. Several other studies showed the effect of curcumin in the inhibition or downregulation of various oncogenes such as EGFR, HER-2, PI3 K/Akt, and MAPK pathway [127–131]. Curcumin is involved in the induction of apoptosis through downregulating the expression of c-myc, Bcl-2, and mutant-type p53, and upregulating the expression of Fas [132].

### 2.10. Effect of Curcumin on Signal Transducer and Activator of Transcription 3 (STAT3)

The Signal Transducer and Activator of Transcription 3 (STAT3) protein is one of the important members of the STAT family of transcription factors [133]. STA3 plays a role in the cancer development and progression and overexpression or high level of STAT3 has been observed in various types of cancers [134, 135]. Curcumin inhibits constitutive STAT3 phosphorylation [136]. Other results also show that the curcumin significantly suppressed Stat3 phosphorylation in bronchoepithelial cells and lung cancer derived cells, indicative of Stat3 pathway suppression, and finally inhibits the proliferative capacity of bronchoepithelial cells and lung cancer cells [137].

### 2.11. Effect of Curcumin in Peroxisome Proliferator-Activated Receptors (PPARs)

PPARs belong to the super family of nuclear receptors, containing three genes that give different subtypes such as PPAR-α, PPAR-*δ*, and PPAR-*γ* [138]. Curcumin showed a role in the upregulation of PPAR-*γ* [139] and interrupted with PDGF and EGF signaling, stimulated gene expression of PPAR*γ*, and thereby plays a role in the inhibition of cell proliferation of activated HSCs [140]. A study of curcumin effects on colon cancer cells confirmed that growth inhibition and stimulation of the transactivating activity of peroxisome proliferator-activated receptor c (PPAR-c), which appears to mediate the suppression of gene expression of cyclin D1 and the epidermal growth factor receptor (EGFR) [141].

### 2.12. Effect of Curcumin on Matrix Metalloproteinases-9 (MMP-9)

Matrix metalloproteinases (MMPs) have been considered as one of the important vital molecules assisting tumor cells during metastasis [142–145]. MMP9, member of the matrix metalloproteinases (MMPs), shows a major role in the breakdown of extracellular matrix in normal physiological processes, including embryonic development, reproduction, and tissue remodeling, as well as in disease processes such as tumor metastasis [146]. Altered expression of MMP-9 has been observed in various types of tumors. However, curcumin shows a vital role in the inhibition of MMP-9 activities and finally plays a role in the management of cancer. A study showed that curcumin inhibits TPA-induced MMP-9 expression and cell invasion through suppressing NF-*κ*B and AP-1 activation [147].

Another study showed that curcumin significantly inhibited the MMP-9 enzymatic activity and protein expression that was induced by PMA [148]. An important study has shown in a human breast cancer xenograft model that administration of curcumin noticeably decreased metastasis to lung and suppressed the expression of NF-*κ*B, MMP-9, COX-2, VEGF, and intercellular adhesion molecule-1 [149]. Earlier results suggest that curcumin plays a role in regulating cell metastasis by inhibiting MMP-2 and MMP-9 in breast cancer cell line [150]. Curcumin showed inhibition of phorbol ester-induced upregulation of cyclooxygenase-2 and matrix metalloproteinase-9 in MCF10A human breast epithelial cells study [151].

## 3. Clinical Trials Based Study of Curcumin

Several valuable clinical trials have been performed using turmeric and its constituents to check their efficacy and safety. Curcumin shows the chemopreventive effect in various types of tumor via modulation of biological processes. An important study based on twenty-five patients with several different premalignant or high-risk lesions suggested that oral curcumin may have chemopreventive effects on the progression of these lesions [152]. Another uncontrolled study based on advanced colorectal cancer refractory to standard treatments, glutathione S transferase, has shown a 59% reduction in the activity with the oral curcumin extract dose of 440 mg daily and five patients maintained radiologically stable disease over the 2- to 4-month study period [153]. Another study in chronic smokers was performed to check the antimutagenic effects of turmeric and it was found that that turmeric significantly reduced the urinary excretion of mutagens in smokers with doses of 1.5 gms/day for 30 days, whereas in the control group (non-smokers), there was no change in the urinary excretion of mutagens after 30 days [154]. An important study was performed in 39 subjects (thirteen with dental caries, twenty-one with head and neck cancer, and five healthy volunteers) and saliva was collected in 50 mL tubes, before and after one hour when subjects chewed two curcumin chaplets. It was found that curcumin treatment led to a reduction in IKK*β* kinase activity in the salivary cells of head and neck squamous cell carcinoma [155]. A study was performed to evaluate the safety and feasibility of combination therapy using curcumin with gemcitabine-based chemotherapy on twenty-one patients and showed 8 gms oral curcumin daily with gemcitabine-based chemotherapy was safe and feasible in patients with pancreatic cancer [156]. Another important study was performed on patients with colorectal cancer and patients were ingested curcumin capsules with a dose of (3.6 g, 1.8 and 4.5 g daily) for 7 days. This finding suggests that a daily dose of 3.6 grams curcumin is pharmacologically effective in the colorectum with negligible distribution of curcumin outside the gut [157].

## 4. Toxicity of Curcumin

Turmeric and its constituents play a vital role in the management of various diseases including cancer. Toxicity and lethal dose level of curcumin are important before using in health management. Several studies were performed to check the safe dose of curcumin in animal model studies. No significant toxicity was observed of turmeric and its constituent curcumin at various doses. An important study in which animals were fed curcumin with a dose of 1.8 gms/kg and 0.8 mg/kg in rat and monkey, respectively, for 90 days showed no adverse effects [158]. An important study in which animals were fed curcumin with a dose of 1.8 gms/kg and 0.8 mg/kg in rat and monkey, respectively, for 90 days showed no adverse effects [158]. Curcumin is remarkably well tolerated, but its bioavailability is poor. It does not show to be toxic to humans [159] even at high doses. Earlier studies concluded that combination therapy using 8 g oral curcumin daily with gemcitabine-based chemotherapy was safe and feasible in patients with pancreatic cancer [156] and other study concluded that oral curcumin is well tolerated and, despite its limited absorption, showed biological activity [160]. An important study based on advanced pancreatic cancer patients showed that 5 patients out of 17 patients receiving curcumin with dose 8 gms/day with gemcitabine showed intractable abdominal pain after a few days to 2 weeks of curcumin intake [161].

A study reported that hepatotoxicity was seen in mice fed with whole turmeric (0.2%, 1%, 5%) or ethanolic turmeric extract (ETE; 0.05%, 0.25%) for 14 days [162]. Earlier report based on curcumin has shown that curcumin doses ranging from 0.45 to 3.6 gms/day for 1 to 4 months showed nausea and diarrhea and also caused an increase in serum alkaline phosphatase and lactate dehydrogenase contents [163].

## 5. Role of Analogue/Derivatives of Curcumin

Some drugs due to their hydrophobic nature show poor bioavailability and a very low quantity of drugs go to the target tissues and show less activity. Low bioavailability of curcumin, due to its low aqueous solubility, has been a major obstacle for its clinical development as a therapeutic drug [164].

However, increasing the absorption of drugs for better activity is the main research of interest in this vista. Analogues or derivatives of drugs show higher absorption and better activity without any adverse complications. Nanoparticle/encapsulation systems give better efficacy and bioavaibility of drugs and also provide the best option in the health management without any untoward effects. Several medicinal plants and their constituents play a significant role in disease management via modulation of various biological activities. But medicinal plants and their constituents show some limitation in the term of efficacy due to the low absorption capacity. Therefore, in clinical trials of oral administration of curcumin to human cancer patients, the systemic availability of curcumin was found to be negligible because of poor absorption of the compound [153, 157].

Clinical trials based studies showed that various types of derivatives such as FLLL 11 and FLLL 12, RL 90, RL91, and GO-YO30 play a significant role as therapeutic drugs.

Earlier investigators showed that GO-Y030, curcumin analogue, inhibited colorectal carcinoma cell growth* in vitro* and in a mouse model [165] and this analogue also inhibits STAT3 activity and cell growth in the breast and pancreatic carcinomas [166]. The inhibition of STAT3 via GO-Y030 also plays an important role in downregulation of the expression of STAT3-regulated genes in colorectal cancer stem cells, such as Cyclin D1 [167], surviving [168], Bcl-2, and Bcl-XL [167, 169]. An important study showed that GO-Y030 reduced the STAT3 downstream target gene expression and induced apoptosis in colon cancer stem cells [170].

Another important study reported that analogues such as GO-Y030 and GO-Y078 showed 7- to 12-fold more potent growth suppression of myeloma cells and showed 6- to 15-fold stronger inhibition of NF-*κ*B, PI3 K/AKT, JAK/STAT3, and IRF4 pathways than curcumin [171]. In MDA-MB-231 cells GOYO30 has a reported IC50 of 1.2 *μ*M [172], significantly less than curcumin.

An important study has compared the inhibitory efficacy of analogues of curcumin such as GO-Y030, FLLL-11, and FLLL-12 in colorectal cancer cell lines including HCT116, HT-29, and SW480 and found that GOY030, FLLL-11, and FLLL-12 showed more potent inhibition of cell viability/proliferation in all HCT116, HT-29, and SW480 human colorectal carcinoma as compared to curcumin [166].

Earlier investigators reported that analogues such as FLLL11 and FLLL12 showed more potency than curcumin at inhibiting cell viability, cell migration, and colony formation in soft agar than curcumin, and theses analogues induced apoptosis in human breast and prostate cancer cells. FLLL11 and FLLL12 analogues of curcumin also synergize with doxorubicin to suppress the growth of MDA-MB-231 breast cancer cells [173]. Earlier investigators showed that D6 compound promotes apoptosis in melanoma cells via the mitochondrial intrinsic pathway [174]. The analogues such as 2,6-bis(pyridin-3-ylmethylene)-cyclohexanone (RL90) and 2,6-bis(pyridin-4-ylmethylene)-cyclohexanone (RL91) modulated the expression of cell signaling proteins, specifically, in SKBr3 cells, protein levels of Her-2, Akt, and NF*κ*B were decreased whereas activity of stress kinases JNK1/2 and P38 MAPK were increased [175].

A recent study showed that RL66 decreased the phosphorylation of Akt on Ser-473 in a time-dependent manner. RL66 decreased Akt phosphorylation after 6 h in MDA-MB-231 cells, whereas the phosphorylation of Akt was only decreased after 36 h in MDA-MB-468 cells [176]. Other studies also reported that RL66 had superior cytotoxicity compared to other analogs of curcumin such as 3,5-bis(flurobenzylidene) piperidin-4-one (EF24) [177], 5-bis(4-hydroxy-3-methoxy-benzylidnen)-N-methyl-4-piperidone [178] (PAC) and GO-Y030 [172] in MDA-MB-231 cells.

## 6. Bioavailability of Curcumin

*Curcumin* shows a vital role in health management through the modulation of various biological activities including regulation of molecular pathways. Therefore, in spite of its potential effects in health benefits of curcumin are limited due to its poor solubility, low absorption from the gut, rapid metabolism, and rapid systemic elimination [179]. Enhancement of absorption, solubility, and slowing down the rapid metabolism of* curcumin* are a main interest of research in medical sciences. In this vista, various studies based on animal model and clinical trials have proved that new formulation of curcumin based on nanoparticles, liposomes, and other new formulations shows a valuable role in health management due to high absorption, solubility, and slowing down the rapid metabolism compared to normal curcumin. However, new formulation of curcumin shows better therapeutic role in health management due to increased or enhanced bioavailability.

Curcumin revealed poor bioavailability has been well recognized by earlier finding [164] and a study reported that 10 mg/kg of curcumin given intravenously to rats yield a maximum serum level of 0.36 ± 0.05 *μ*g/mL, whereas 500 mg/kg of curcumin administered orally only yielded a 0.06 ± 0.01 *μ*g/mL maximum serum level [180].

Furthermore, curcumin was given orally to rats at a dose of 2 g/kg, a maximum serum concentration of 1.35 ± 0.23 *μ*g/mL was observed at time 0.83 hours, while in humans the dose of 2 g of curcumin resulted in either undetectable or very low (0.006 ± 0.005 *μ*g/mL at 1 h) serum levels [181].

Therefore, new formulation based on adjuvants, nanoparticles, liposomes, micelles, and phospholipid complexes is currently evaluated/used to increase the bioavailability and biological activity of curcumin[182–186]. An important study formulated innovative preparation such as THERACURMIN and confirmed that its oral bioavailability is approximately 30-times higher than curcumin powder in both rats and humans and results also show that THERACURMIN enhanced gastrointestinal absorption as a result of colloidal dispersion [187]. Another study was performed to evaluate the safety and pharmacokinetics of newly developed nanoparticle curcumin with increased water solubility such as THERACURMIN and it was concluded that THERACURMIN can safely increase plasma curcumin levels in a dose-dependent manner at least up to 210 mg without saturating the absorption system [188]. A study also reported that curcumin loaded cellulose nanoparticles (cellulose-CUR) formulation showed the highest cellular uptake and caused maximum ultrastructural changes related to apoptosis in prostate cancer cells [169] and other study concluded that SF-derived curcumin nanoparticles show higher efficacy against breast cancer cells and also have the potential to treat* in vivo* breast tumors [189].

A study was performed to evaluate the liposomal curcumin's potential against cancer models of mesenchymal (OS) and epithelial origin and it was observed that 2-Hydroxypropyl-*γ*-cyclodextrin/curcumin-liposome complex shows promising anticancer potential both* in vitro* and* in vivo* against KHOS OS cell line and MCF-7 breast cancer cell line [190].

A study based on* in vivo* pharmacokinetics showed that curcumin entrapped nanoparticles demonstrate at least 9-fold increase in oral bioavailability as compared to curcumin administered with piperine as absorption enhancer [191] and study based on human colorectal cancer cell lines such as LoVo and Colo205 cells showed that* in vitro* treatment with liposomal curcumin induced a dose-dependent growth inhibition and apoptosis [192]. An important study showed that encapsulating the curcumin into the hydrogel nanoparticles yielded a homogenous curcumin dispersion in aqueous solution as compared to the free form of curcumin [193] and earlier finding observed that after oral administration of CUR-PLGA-NPs, the relative bioavailability was 5.6-fold increased and also showed longer half-life compared with that of native curcumin. Increased oral bioavailability of CUR may be linked with improved water solubility, higher release rate in the intestinal juice, enhanced absorption by improved permeability, inhibition of P-glycoprotein- (P-gp-) mediated efflux, and increased residence time in the intestinal cavity [194]. An important study has discussed in detail regarding the most recent development in bioavailability, absorption, and metabolism of curcumin in detail [195].

## 7. Conclusions

Cancer is a deadly disease for both men and women and also a major health problem worldwide. The present mode of treatment based on chemotherapy and radiotherapy is very expensive and also exhibits serious side effects in human beings. Keeping in view the significance of herbs, this review is written to show the role of curcumin in the prevention of various types of cancer through the activation or inactivation of various genetic pathways. These reported features combined with the absence of side effects and being inexpensive as well as easy to access, turmeric and its constituent curcumin may be proved very effective therapeutics in the management of cancers.
